# Enhanced cardiac vagal tone in mental fatigue: Analysis of heart rate variability in Time-on-Task, recovery, and reactivity

**DOI:** 10.1371/journal.pone.0238670

**Published:** 2021-03-03

**Authors:** András Matuz, Dimitri van der Linden, Zsolt Kisander, István Hernádi, Karádi Kázmér, Árpád Csathó

**Affiliations:** 1 Department of Behavioural Sciences, Medical School, University of Pécs, Pécs, Hungary; 2 Department of Psychology, Education, and Child Studies, Erasmus University Rotterdam, Rotterdam, The Netherlands; 3 Institute of Information and Electrical Technology, Faculty of Engineering and Information Technology, University of Pécs, Pécs, Hungary; 4 Department of Experimental Neurobiology, University of Pécs, Pécs, Hungary; 5 Szentágothai Research Center and Center for Neuroscience, University of Pécs, Pécs, Hungary; University of Massachusetts Boston, UNITED STATES

## Abstract

Heart Rate Variability (HRV) has been suggested as a useful tool to assess fatigue-sensitive psychological operations. The present study uses a between and within-subject design with a cognitively demanding task and a documentary viewing condition, to examine the temporal profile of HRV during reactivity, Time-on-Task (ToT), and recovery. In the cognitive task group, participants worked on a bimodal 2-back task with a game-like character (the Gatekeeper task) for about 1.5 hours, followed by a 12-minute break, and a post-break block of performance (about 18 min). In the other group, participants watched documentaries. We hypothesized an increasing vagal-mediated HRV as a function of Time spent on the Gatekeeper task and no HRV change in the documentary viewing group. We also analyzed the trial-based post-response cardiac activity as a physiological associate of task-related motivation. Relative to the documentary-viewing, ToT was associated with an elevated level of subjective fatigue, decreased heart rate, and increased HRV, particularly in the vagal-mediated components. Based on fatigued participants’ post-error cardiac slowing, and post-error reaction time analyses, we found no evidence for motivation deficits. The present findings suggest that the parasympathetic branch of the autonomous nervous system functioning as a relaxation system tends to be activated under increasing mental fatigue. In addition, the study shows that many HRV indices also seem to change when individuals are engaged in a prolonged, less fatiguing activity (e.g. documentary viewing). This finding emphasizes the relevance of comparisons/control conditions in ToT experiments.

## Introduction

Mental fatigue induced by the prolonged performance of a cognitively demanding task (i.e. Time-on-Task) has a detrimental effect on a wide range of prefrontal-cortex loaded cognitive functions, reduces the willingness to exert further effort, and is frequently accompanied with reduced performance efficiency and an increased number of errors (e.g. [1–3]). The autonomic nervous system as a neurophysiological substrate of adaptive modulation of behavior under changing environmental conditions is known to have a vital role in the performance of cognitively demanding, and prolonged tasks (e.g. [4, 5]). From this perspective, it is understandable why several previous studies have explored the association between mental fatigue and Heart Rate Variability (HRV). HRV is an index of cardiac autonomic regulation (i.e. the variability in intervals between successive heartbeats) that can be linked to many brain areas [6] and various psychological phenomena including many that have also been associated with fatigue. More specifically, of the different HRV components, the vagal mediated components seem to be associated with structural variations in the striatal and limbic structures suggesting that these brain areas may serve as important anatomical basis of parasympathetic autonomic modulation [7].

Accordingly, HRV is assumed to be an ideal tool to examine the association between fatigue-vulnerable psychological operations and autonomic processes. Previous studies examining the HRV–fatigue associations indeed converged on the conclusion that HRV is a significant associate of fatigue that can predict the concurrent drop in cognitive performance due to Time-on-Task (see e.g. [8–13]).

Nevertheless, as fatigue is a complex, multifaceted state and HRV has many calculable components that have diverse sources, this greatly complicates the exact biopsychological interpretation of the HRV–fatigue associations. Therefore, in recent studies, it has been emphasized that for better comparability, future studies should build on more recent insights regarding the physiological and methodological substrates of HRV [14, 15]. Specifically, it has been suggested that, instead of analyzing HRV indices under a mixed effect of parasympathetic and sympathetic activity (e.g. the ratio of the low and high frequency components of the HRV spectrum), the studies should focus mainly on those HRV indices that clearly reflect parasympathetic control on cardiac activity (i.e. vagal tone) and, therefore, have a clear functional interpretation. Cardiac vagal tone as an index of the parasympathetic nervous system activity predicts a broad range of cognitive functions [16] that are vulnerable to Time-on-Task such as executive [17], and attentional functions [18], working memory [19], and emotion regulation [20]. Importantly, it has been suggested that increased parasympathetic activation (i.e. increased vagal tone) implies that participants disengaged from the task at hand [21]. Task disengagement is considered to be one of the strongest behavioral manifestations of mental fatigue [22, 23]. Therefore, it is relevant to analyze cardiac vagal tone in the context of fatigue induced by Time-on-Task.

Given the above findings and recommendations, in the present study, we hypothesized that the vagal components of HRV are predictive for fatigue related changes, that is, the root mean square of successive differences (RMSSD), the percentage of interbeat intervals that differ by more than 50 ms (pNN50), and the high frequency (HF) component of HRV. RMSSD and pNN50 are the primary time-domain measures used to estimate vagal-mediated changes and are relatively free of respiration signal components [24, 25]. The HF component also reflects vagal tone, but it more strongly corresponds to the respiratory cycle [15, 25].

In a recent study, we found evidence for increased vagal-mediated HRV with increasing Time-on-Task on a bimodal task-switching task [26]. An important limitation of that HRV measurement, however, was the lack of comparison with a cognitively less demanding condition. Therefore, in the current study, we compared HRV in two different groups: In a group in which participants had to engage in a cognitively demanding bimodal 2-back task (i.e. Gatekeeper task group [27, 28]; and a Documentary-viewing group in which participants watched emotionally neutral documentary films without any specific task. The 2-back task chosen was the recently developed Gatekeeper task [27, 28]. The Gatekeeper task is a bimodal 2-back task that requires individuals to decide whether, based on their memory, the actual pair of stimuli (visual and auditory) is identical to the pair of stimuli occurred two trials earlier. The suppression of interference between the trials and the two modality channels require a high level of cognitive control. Specifically, it is known to put high demands on working memory (keeping information active over time, and updating). As such, we expected that the cognitive demands associated with the Gatekeeper task would be well suited to induce mental fatigue. In contrast, the documentary film viewing has been proven to be cognitively less demanding and, therefore, has been used as a control condition in many previous fatigue studies [29–32].

Following the line of reasoning above, in the Gatekeeper task group, we hypothesized an increasing vagal-mediated HRV (i.e. increasing RMSSD, HF, and pNN50 components) as a function of Time-on-Task. In contrast, we expected to find no change in vagal mediated HRV in the Documentary-viewing group.

In designing the study, we followed the recommendation of Laborde and colleagues [15] by testing the changes in HRV in the resting and active phases of the experiment using the “three Rs” concept: resting, reactivity, and recovery. Specifically, in addition to the change in HRV while performing the task for a prolonged period (i.e. Time-on-Task), we also explored the change from active task performance to a resting state (recovery) and, conversely, from a resting state to active performance (reactivity). In order to assess the recovery-related effects, the last block of trials in our study was preceded by a break period of 12 minutes. We argue that by investigating the reactivity and recovery related changes in HRV we can identify more clearly the changes in vagal tone in an initial task performance phase and in a phase when individuals became fatigued. Such changes in vagal activity may provide insight into the flexibility of parasympathetic activation which was found to be essential to stabilizing performance in demanding cognitive tasks [33]. With other words, in addition to examining changes during Time-on-Task, the reactivity and recovery related changes in the vagal mediated HRV may indicate the sensitivity of the parasympathetic system to alterations in task demands. Our theoretically framework on the association between vagal mediated HRV and fatigue does not necessarily include hypotheses for the reactivity and recovery related changes in HRV. Therefore, our approach regarding the reactivity and recovery effects is exploratory.

Finally, one reoccurring topic in fatigue research is the extent to which the effects of Time-on-Task reflect fatigue instead of a mere loss of interest or willingness to keep on doing one’s best during the task. There is now a great number of evidence supporting the latter point, that is, that persons’ motivation or willingness to exert further effort (task-engagement) during a prolonged task performance seems to be an essential factor regarding both the subjective state of fatigue and the concordant performance decrements [22]. In this concept, mental fatigue is an urge for relaxation or recovery in order to prevent the person from too much effort exerted in a task with low expected value of the outcomes [3]. In line with this motivational account of fatigue, many empirical studies confirmed that after being presented with reward, fatigued participants remarkably improved their performance [23, 34, 35]. In one study, such recovery was found to be accompanied with a lowered level of HRV [36]. This is also in line with the finding of Pattyn et al. [21] that a relaxed autonomic system expressed by an increased vagal tone predicted that participants became disengaged from the performance of the task. Alternatively, a decreased vagal tone may provide evidence for an enhanced compensatory effort that participants made against the potential impairment of task performance caused by fatigue [10].

In addition to the associations found between HRV and task-(dis)engagement, we also aimed to gain insight into the participants’ motivational stance based on the comparison of phasic cardiac activity and reaction times after accurate and inaccurate responses. Specifically, several lines of evidence suggest that phasic heart rate deceleration [37] and reaction time slowing after an inaccurate response are correlates of general performance monitoring and error awareness [22]. Post-error heart rate deceleration was found even if no feedback was given about correctness suggesting that changes in phasic heart rate is not only a feedback-related reaction (i.e. feedback valance related), but also a part of a general performance monitoring mechanism [37]. Performance monitoring has been found to show a decline with increasing mental fatigue in relation to individuals’ decreased motivation to perform the task [22]. These earlier findings underline the relevance of analyzing changes in post-response phasic cardiac activity and post-error slowing in reaction times in the current study. Importantly, to our knowledge, no previous study has addressed post-response cardiac activity in fatigue research.

To summarize, in the present study, we aimed to examine the temporal profile of HRV, including the changes related to Time-on-Task, recovery, and reactivity. We hypothesized that the mental fatigue state is accompanied with an increased vagal-mediated HRV as a function of Time-on-Task. We also argue, however, that the examination of reactivity- and recovery-related changes are important to draw more robust conclusions about how parasympathetic control on cardiac activity associated with the performance of fatiguing, cognitively demanding tasks. In addition we analyzed the trial-based post-response cardiac activity and post-error slowing in reaction times as physiological and psychological correlates of performance monitoring.

## Materials and methods

### Participants

Forty-four participants (under- and post-graduate students), in a medication-free health condition, with normal hearing and normal or corrected-to-normal vision participated in the study. There were 22 participants in the Gatekeeper task group and 22 participants in the Documentary-viewing group. Due to technical failures, the data of three participants were excluded from the analyses. Thus, the final dataset contained data from 20 participants (11 females, mean age: 21.2 with *SD* of 2.21, range: 19–27) in the Gatekeeper task group and 21 participants (11 females, mean age: 22.5 with *SD* of 3.9, range: 18–29) in the Documentary-viewing group. Participants in the two groups were matched in age (*t*(39) = - 1.33, *p* = .19, *d* = .42) and gender (*χ*^2^ = .22, *p* = .64). All participants provided written consent. The study meets ethical standards according to the Declaration of Helsinki and was approved by the Ethics Committee of the University of Pécs (nr. 7698).

The minimum sample size to ensure the statistical power of the main effect of Time-on-Task on HRV was estimated based on our recent study [26], as well as other recently published studies that examined active task-performance and a resting periods or modulation of HRV by time on task(e.g. [8, 38–40]). By applying the lowest effect size reported in Matuz et al. [26], which is a *η*_p_^2^ of 0.14 for the high-frequency HRV component, the recommended minimum sample was 28 participants to achieve a power level of 90% at an alpha < 0.05 (by Gpower 3.1., [41]). For the interaction effects (i.e. Group x Time-on-Task), sample size calculation was based on the effect sizes reported in Hidalgo-Muñoz et al. [38], because this study maps closest to our design by comparing low-, and high cognitive workload conditions under fatigue. By applying the lowest effect size for interaction (i.e. a *η*_p_^2^ of 0.23 for the pNN50 index), the minimum sample size was 18 to achieve a power level of 90% at alpha < 0.05. This was further supported by the fact that the total sample sizes in psychophysiological studies with similar aims and design ranged between 20 and 37 [32, 42, 43]. Participants’ age and education background as well as the statistical tests performed in the previous studies were highly similar to those in the current study. To sum up, the final dataset of 41 participants had the appropriate statistical power to detect main effects as well as the interactions we aimed to examine.

### Task and stimuli

#### Gatekeeper task

**Fig 1:**
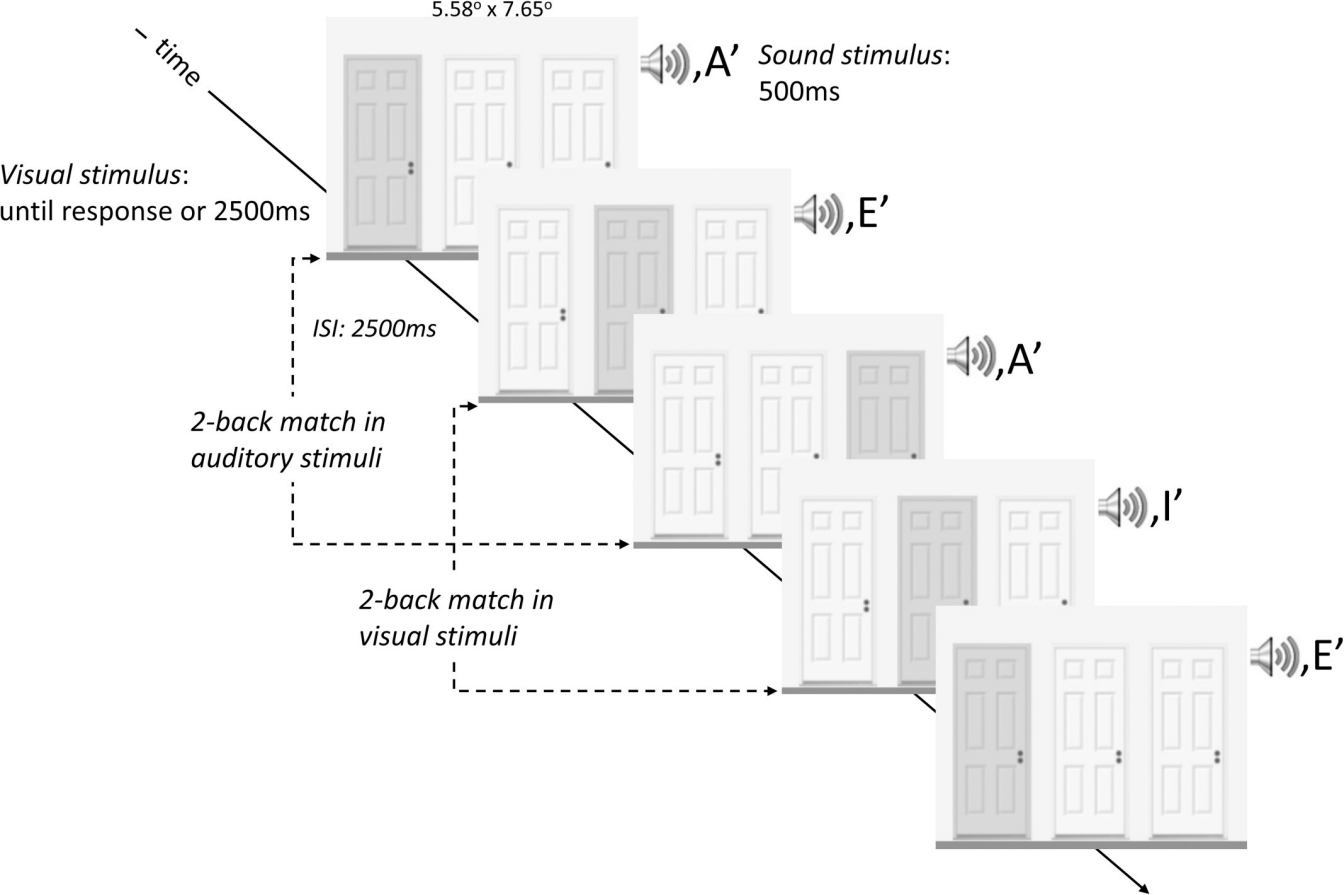
Schematized sequence of trials in the Gatekeeper task. The Gatekeeper task is a dual 2-back task with visual and auditory stimuli. The Gatekeeper task has a game-like character: participants need to imagine that they are a nightclub doorperson and need to memorize the door and the password used by the guests of the club for entry. In each trial, the visual stimuli (i.e. three door images, one of which is highlighted in red) and the auditory stimulus (i.e. one spoken letter) were presented simultaneously. For dual target condition, both the visual and auditory stimuli were identical to those presented two trials earlier. For the single target conditions, a 2-back match occurred either for the auditory stimulus (single auditory target condition) or for the visual stimulus (single visual target condition). For the no target condition, both the visual and the auditory stimuli were different to the stimuli shown two trials earlier. In each trial, participants were instructed to indicate by pressing a key whether there is a 2-back match in any modality.

Participants in the Gatekeeper task group performed an adapted version of the Gatekeeper task from Heathcote et al. [27, 28] which is a dual 2-back task with visual and auditory stimuli. The Gatekeeper task has a game-like character: participants need to imagine that they are a nightclub doorperson and need to memorize the door and the password used by the guests of the club for entry. This game-like feature of the task is an asset because it is expected to enhance task engagement, which may lead to less boredom during Time-on-Task. In each trial, the visual stimuli (i.e. three door images, one of which is highlighted in red) and the auditory stimulus (i.e. one spoken letter) were presented simultaneously (see Fig 1). Four different stimulus conditions were prepared. For dual target condition, both the visual and auditory stimuli were identical to those presented two trials earlier. For the single target conditions, a 2-back match occurred either for the auditory stimulus (single auditory target condition) or for the visual stimulus (single visual target condition). For the no target condition, both the visual and the auditory stimuli were different to the stimuli shown two trials earlier. In each trial, participants were instructed to indicate by pressing a key whether there is a 2-back match in any modality, which in this case would imply that the ‘guest’ would not be allowed to enter the night club. In the instructions, it was emphasized that both speed and accuracy are equally important. No feedback was given about the correctness of the response. A new trial began after a 2.5s interval after response.

#### Documentary film viewing

The participants in the Documentary-viewing group watched three documentary films (about 30 minutes each) for 1.5 hours: Planet Earth Episode 7 Great plains (2007); When we left Earth–The NASA missions: The Shuttle (2008); and Ocean oasis (2000) (see also a recent study by Takács et al. [32]. The films were presented in a counterbalanced order across participants. A few emotionally arousing scenes were cut from the documentaries without creating strange transitions in the narrative actions.

### Procedure

**Fig 2:**
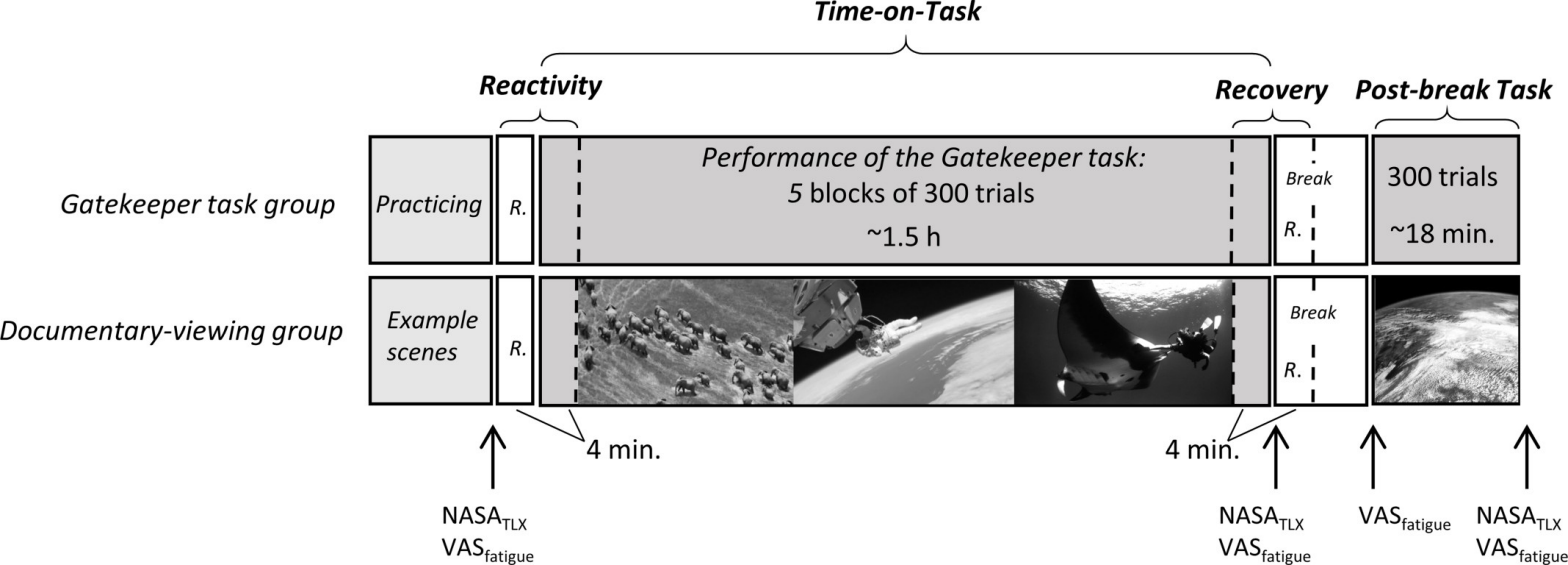
The schematized procedure of the study in the Gatekeeper task group and Documentary-viewing group. Participants in both groups had a 4-minute resting period before the Time-on-Task when ECG was recorded. It was followed by the Time-on-Task period, when the participants in the Gatekeeper task group performed 5 blocks of 300 trials of the Gatekeeper task without rest. The participants in the Documentary-viewing group watched documentaries. ECG was continuously recorded during the Time-on-Task in both groups. After that, participants had a break of 12 minutes. During the break, a 4-minute ECG was recorded again. Then, an additional block of 300 trials were administered for the Gatekeeper task group and documentaries were presented for the Documentary-viewing group. R: resting ECG. Participants indicated their subjective fatigue and workload multiple times during the experiment.

Fig 2 schematizes the procedure of the experiment in the two groups. Participants were asked to get adequate sleep during the night prior to the experiment and to abstain from alcohol and caffeine-containing substances before the experiment. In addition, they were told that they should avoid exhausting physical and mental activities (e.g. physical workout, studying for a class) before the experiment. Participants’ sleep duration was monitored using an actigraph (Gatekeeper task group: 7.46h, SD = 1.64h; Documentary-viewing group: 7.82h, SD = 1.48h) and by self-reporting (Gatekeeper task group: 7.67h, SD = 1.61); Documentary-viewing group: 7.82h, SD = 1.48). Participants in the two experiments did not significantly differ in self-reported sleep (t(39) = 1.15, *p* = .26, *d* = .36) or in the actigraph data (t(39) = —.70, *p* = .49, *d* = .23).

#### Gatekeeper task group procedure

The experimental sessions started between 9:30 a.m. and 13:30 p.m. Participants were seated in a soundproof and uniformly air-conditioned (23C^o^) laboratory. Following the task explanation, the electrocardiographic (ECG) electrodes were set up (three chest electrodes, Lead II.). Then, the participants performed *72* practice trials. None of the participants indicated sensory irritability or unpleasantness after the practice trials. In addition, participants filled in two scales regarding the subjective task load and fatigue (see Subjective Measures section).

After the completing the self-reported measures, a short adaptation period followed (3 minutes), in which participants remained rested and sit comfortably. After that, a 4-min-long resting ECG was recorded (with eyes open, sitting with knees at about a 90° angle, the feet flat on the floor). Then, the prolonged performance of the task followed. During this Time-on-Task period of the Gatekeeper task the participants performed *5* blocks of 300 trials without rest. The exact duration of this phase depended on the participants’ response time, and thus slightly varied across participants (average duration total Time-on-Task: 1.48 h, range: 1.31–1.67 hours, *SD* = 0.1 h). When the 5 blocks of trials were completed, participants again filled in the subjective measures. Subsequently, participants had a break of 12 minutes. During the first 4 minutes of this break period, resting ECG was recorded. After the break, participants were asked to indicate their fatigue level again. Then, an additional block of 300 trials were administered, after which the participants had to fill in their fatigue level and perceived load during the last block.

#### Documentary-viewing group procedure

In the Documentary-viewing group, the procedure was largely identical to that of the Gatekeeper task group. Participants first had a familiarization period by being shown some example scenes from the documentaries (these scenes were not presented later in the viewing period). Participants were then instructed to watch the films, but it was emphasized that they did not have a particular task to perform (e.g. they did not need to memorize information provided by the documentaries). The ECG recording and the administration of the self-reported measures (fatigue, task load) followed the same procedure as in Gatekeeper group. The only difference was that we reduced the number of scales to assess participants’ subjective workload: participants reported only the perceived mental and physical demand and their frustration level.

### Self-reported measures of fatigue and workload

In each group, participants completed the NASA Task Load Inventory (NASA_TLX_; [44]) three times: after the practice (i.e. in the Documentary-viewing group, after watching examples documentary scenes); after Time-on-Task period; and after the post-break task. The NASA_TLX_ is a multidimensional self-reported measure to assess individuals’ perceived workload during the task on *6* scales with 21 gradations: mental demand; physical demand; temporal demand; overall performance level; effort; and frustration level. Participants were also asked to indicate the level of fatigue they experienced on a Visual Analogue Scale (VAS_fatigue_; 100mm long line, “No fatigue at all” was printed on the left side and “Very severe fatigue” on the right side).

### Heart rate variability measurement

*ECG* data were digitized at a sampling rate of 1 kHz at 16-bit resolution with a CED 1401 Micro II analogue-digital converter device (CED, Cambridge, UK). The ECG signals were visually inspected, and artefacts were corrected, and if necessary removed. Subsequently, participants’ R-R intervals, in milliseconds, were extracted using Spike2 software. The time elapsed between two successive R-waves (R-R intervals) were analyzed further by Kubios HRV analysis package 2.0 [45]. The artefacts within the R-R intervals were again corrected using the low artefact correction option of the Kubios software: detected artefact beats were replaced using cubic spline interpolation. Frequency-domain, time-domain, and non-linear HRV measures were calculated.

The frequency indices included the absolute high frequency power (0.15Hz—0.4 Hz; ms^2^; HF), the log-transformed high frequency power (_ln_HF), the absolute low frequency power (0.04Hz– 0.15Hz; ms^2^; LF), and the log-transformed low frequency power (_ln_LF). The time-domain measures included the mean heart rate (HR, beats/min), the root mean square of successive differences (RMSSD, ms), the log-transformed RMSSD (_ln_RMSSD), and the percent of the number of pairs of adjacent RR intervals differing by more than 50 ms (pNN50; %). The non-linear measures included the short-term HRV as a measure of the width of the Poincaré cloud (SD1), and the long term HRV as a measure of the length of the Poincaré cloud (SD2). Nevertheless, Ciccone et al. [46], demonstrated that RMSSD and SD1 are both mathematically and empirically identical indices of HRV (i.e. SD1 equals to RMSSD multiplied by 1/ √2). In line with this, each analysis in the current study returned identical results for RMSSD and SD1 up to the third decimal. Therefore, below we do not report the results for SD1. Recently, there has been a growing number of studies suggesting that HRV should be normalized with respect to average heart rate [47–50]. Therefore, we also calculated and analyzed R-R normalized HRV indices. These analyses provided the same conclusions as those without RR normalization. The methods and the results of the R-R normalized analyses are shown in the (S8 and S9 Tables in S1 File).

We used two different intervals for the calculation of each HRV index: 4-minute intervals; and 15-minute intervals. The 4-min intervals were the resting period before the experiment, the first 4 minutes of the first experimental block, the last 4 minutes of the fifth experimental block, the resting period during the break, and, finally, the first 4 minutes of the post-break task block. These short intervals were used in the analysis of the reactivity and recovery effects (see Data Analysis section below). Studies addressing reactivity and recovery related changes in HRV often use even shorter intervals [51–53]. To calculate HRV within the experimental blocks, we selected the middle 15 minutes in each block. Please note that blocks lasted about 18 minutes each but were not completely identical in terms of duration which depended on the participants’ reaction time. We also calculated and analyzed HR and HRV indices in the Time-on-Task with variable block intervals (i.e. full-length blocks), and these results are shown in the (S7 Table in S1 File). The conclusions are identical regardless of whether the analyses were performed for identical intervals (i.e. 15-min) or for the full-length blocks.

In the resting period before the experiments, there were no significant differences between the Gatekeeper task group and the Documentary-viewing group in heart rate (HR) and HRV variables (*p* = .12 - .94; *Cohen’s d* = .02 - .50).

In addition to the HRV measures, for each trial, post-response cardiac activity was also calculated as the average difference in the R-R intervals during the 2.5s-long post-response period. The larger average difference reflected a slower activity after response.

### Data analysis

Statistical analyses were performed by SPSS version 25 (the data and the script for our analyses are available publicly in a data repository: http://dx.doi.org/10.17632/b3svkcpm5d.1; doi: 10.17632/b3svkcpm5d.1). To control for baseline levels of cardiac parameters and pre-Time-on-Task scores on the self-reported measures, we followed the guidelines of Van Breukelen [54, 55] and thus, analyses of covariance (ANCOVAs) were conducted.

Specifically, to analyze the Time-on-Task related changes in subjective fatigue, we performed an ANCOVA with measurement of subjective fatigue after the Time-on-Task period as the dependent variable, Group (Gatekeeper vs. Documentary) as a fixed factor, and the pre-Time-on-Task measure as a covariate. Similarly, we tested the post-break fatigue level as the dependent variable, Group as a fixed factor and post Time-on-Task fatigue as a covariate. Finally, the fatigue effect of the last block (i.e. post-break block) was tested with fatigue after the post-break block as the dependent variable, Group as a fixed factor, and fatigue before the post-break block as a covariate. The same ANCOVA procedure was used for the workload measures.

For the cardiac parameters, we followed the same ANCOVA procedure, in which we included the relevant measurement as dependent variable and the pre-measurement as covariate. In each of these analyses, Group was a fixed factor. We conducted ANCOVAs in this way for reactivity (pre = pre-experiment HRV, post = first 4 minutes of the Time-on-Task period), recovery (pre = last 4 minutes HVR of post = 4-minute HRV during the break), reactivity after break (pre = 4-minute HVR during break, post = first 4 minutes of the post-break block). For the analysis of Time-on-Task related changes in HRV, mixed ANCOVAs were performed with Block (i.e. the first to the fifth block) as a within-subject factor, Group as a between-subject factor and pre-experiment resting HRV as a covariate. The associations between subjective fatigue measured after the Time-on-Task and the changes in HRV during the Time-on-Task were investigated by partial correlation analyses adjusted for pre-experiment fatigue.

Although ANCOVA is suggested as the most appropriate method to perform baseline adjustment in randomized studies, there is still a debate on whether the change score from the baseline should also be used to evaluate pre-post differences (see e.g. [56]). Therefore, the conducted parallel analyses in which we addressed the same questions as above, but test them with ANOVAs of change scores (i.e. using the difference between pre-test and post-test measures as a dependent variable). We report these results in the S1 File. Except for a very few minor cases (indicated in the Results section) the change-score analyses yielded the same conclusions as those with the ANCOVAs.

Finally, to assess the cognitive performance in the Gatekeeper task, reaction times on correct responses (RT) and target-sensitivity (i.e. *d*’; Zhit−Z_false alam_) were calculated for each block and target type. The analysis of cognitive performance is relevant in the present study because it provides information about how fatigue and workload as well as the changes in HRV were concordant with the objective performance. Combined scores of *d*’ and RT (i.e. Z_d’_−Z_RT_) were computed first and then subjected to repeated measures ANOVAs (*r*ANOVAs) with Block (5 blocks of trials) and Target types as within-subject factors. A separate *r*ANOVA was performed to analyze the break-related effects (changes from block 5 to the post-break block).

The Greenhouse–Geisser (ε) adjustment was applied if sphericity was violated. Significant main effects and interactions were followed-up by simple effects analysis using Bonferroni corrections. Age is known to modulate HRV. Therefore, we tested the potential modulatory effect of age on our results. Specifically, we reran each of our analyses with age as a covariate. None of these analyses, however, returned different conclusions as those without the age as a covariate, and the interactions with age were far from significance.

## Results

### Subjective fatigue and workload

The Gatekeeper and Documentary groups did not significantly differ in fatigue before the experiment (t(39) = -1.51, *p* = 0.14, *d =* .47). However, compared to the Documentary-viewing group, participants in the Gatekeeper-task group became more fatigued by the end of the Time-on-Task (F(1,38) = 11.24, *p* < .01, η_*p*_^2^ = .23), but there was no further change in subjective fatigue during the break and in the post-break block. For the workload measures, before the experiments, participants particularly indicated that the Gatekeeper task has high mental demands and requires relatively much effort. The documentary viewing was rated significantly less mentally demanding and less frustrating than the Gatekeeper task (Mental demand: t(39) = 6.89, *p* < .001, *d* = 2.15; Frustration: t(39) = 5.75, *p* < .001, *d* = 1.80). After the Time-on-Task period, workload increased more in the Gatekeeper-task group than in the Documentary-viewing group (Mental demand: F(1,38) = 50.89, *p* < .001, η_p_^2^ = .57; Physical demand: F(1,38) = 31.21, *p* < .001, η_p_^2^ = .45; Frustration: F(1,38) = 12.87, *p* < .001; η_p_^2^ = .25). After the post-break block no significant Group effects were found with ANCOVA (Mental demand: *F*(1,38) = 1.55, *p* = .22, *ηp*^*2*^ = .04; Physical demand: *F*(1,38) = .17, *p* = .69, *ηp*^*2*^ = .00; Frustration: *F(*1,38) = .57, *p =* .46; *ηp*^*2*^ = .02). In contrast, the ANOVAs with change scores indicated reduction in perceived mental demand, physical demand and frustration in the Gatekeeper task group, but not in the Documentary-viewing group (see the S2 Table in S1 File). To summarize, the analyses of subjective fatigue and workload data suggest that the fatigue and workload manipulation was successful. Descriptive statistics for subjective fatigue and workload are presented in the (S1 Table in S1 File).

### Cognitive performance on Gatekeeper task

**Fig 3:**
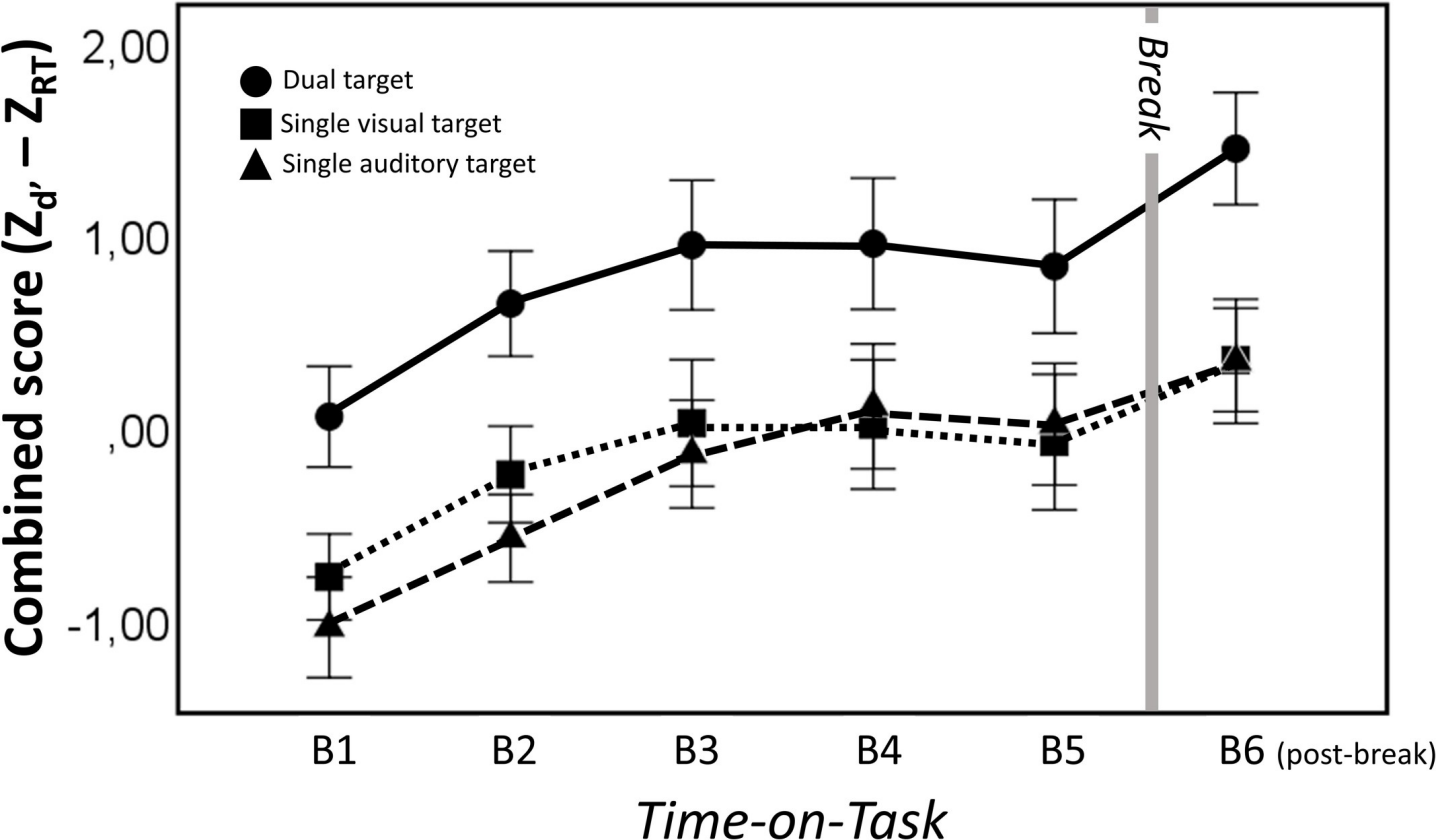
Results of composite scores (Zd’–ZRT) in the Gatekeeper task group. For dual target condition (circle), both the visual and auditory stimuli were identical to those presented two trials earlier. For the single auditory target condition (triangle), a 2-back match occurred for the auditory stimulus. For the single visual target condition (square), a 2-back match occurred for the visual stimulus. Error bars represent SEM.

The main results of the cognitive performance in the Gatekeeper task are depicted in Fig 3 (for descriptive statistics see the, S3 Table in S1 File). The main effect of Block (1 to 5) reached significance for the composite score scores (F(4,38) = 9.57, *p* < .001, η_*p*_^2^ = .34). The corrected post-hoc analysis revealed that participants’ sensitivity to the targets increased from the first to the third block, but showed no significant change thereafter (block 1 vs. block 3: *p* < .01; block 3 vs. block 5: *p =* 1.0). Accordingly, the findings suggest that, despite the strong increase in subjective fatigue and task load, there was no direct decline in performance on the Gatekeeper task. Note, however, that we did find a highly significant improvement in performance after the break (block 5 vs. post-break block: F(1,19) = 10.74, *p* < .01, η_*p*_^2^ = .36), suggesting that performance before the break may nevertheless have been suboptimal.

### Changes in HR and HRV in reactivity

**Fig 4:**
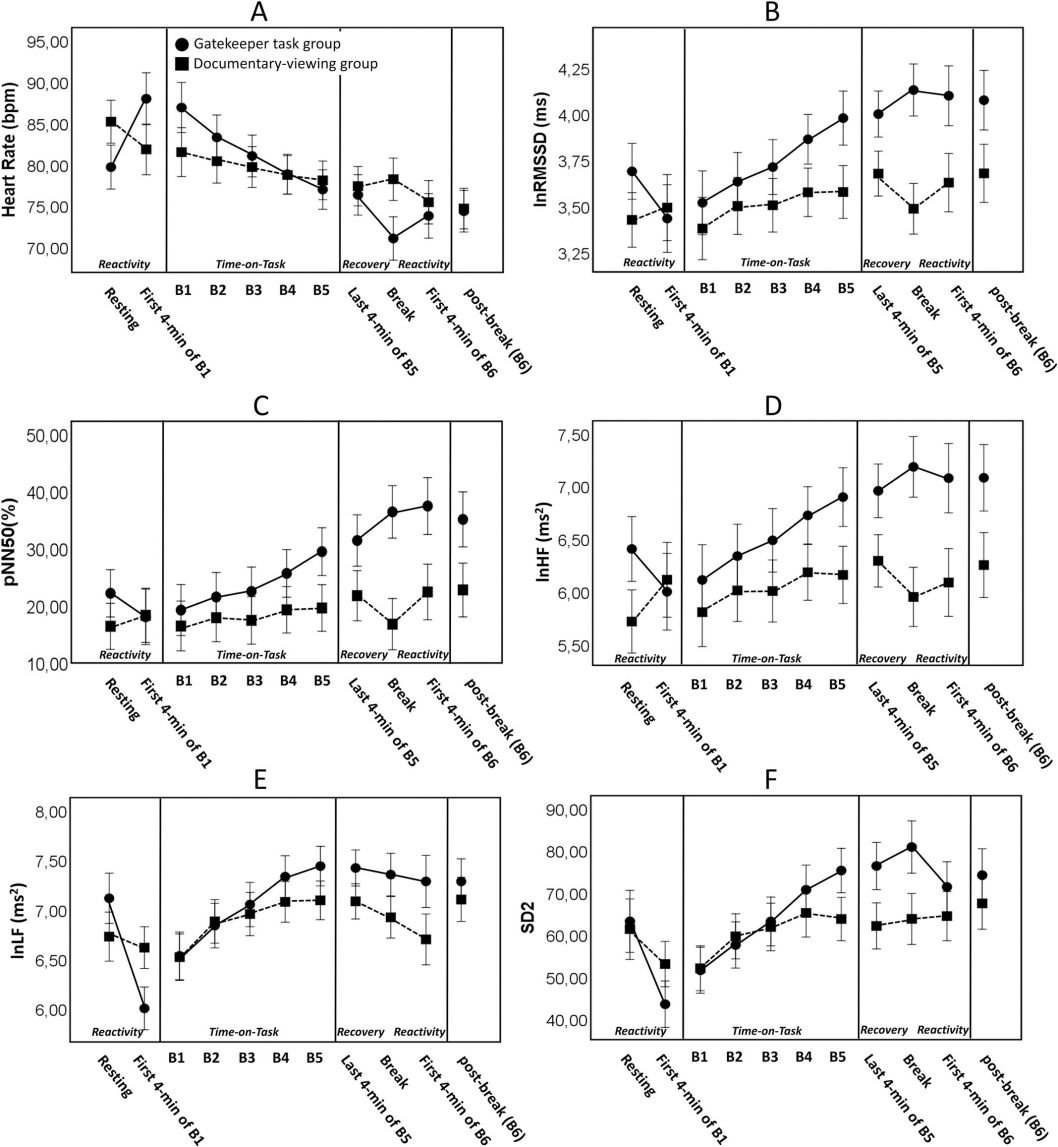
Results of mean heart rate (A) and five heart rate variability measures (B-F) in the Gatekeeper task (circle) and the Documentary-viewing (square) group. The figure presents the results for HRV measures with the most robust Block x Group interaction. Error bars represent SEM. (lnRMSSD: the log-transformed root mean square of successive differences; pNN50: percent of the number of pairs of adjacent R-R intervals differing by more than 50 ms; lnLF; log-transformed Low frequency power; lnHF: the log-transformed High frequency power; SD2: the length of the Poincaré cloud.

Reactivity analyses focused on the changes in HR and HRV from the pre-experiment resting interval to the first 4 minutes of block 1. Table 1 presents the results of the analyses, and Fig 4 depicts the results for the HR and for HRV indices from each domain (for descriptive statistics see the, S4 Table in S1 File). The analysis revealed that HR in the first 4 minutes of the first experimental block was significantly higher in the Gatekeeper task group compared to the Documentary-viewing group. Regarding the specific components of the HRV, SD2 (only with ANCOVA, but not with ANOVA of change scores; see, S6 Table in S1 File), and _ln_LF as well as two vagal-mediated HRV components (i.e. _ln_RMSSD, _ln_HF) were found to be reduced in the Gatekeeper task group in reactivity. This latter finding suggests that reactivity was associated with a significant withdrawal in vagal activity.

**Table 1:** Results of ANCOVAs for the changes in heart rate and HRV in reactivity.

Variables	Analysis		
	Group effect		
	F_(1,38)_	*p*	η_*p*_^2^
HR	39.93	< .001	.51
RMSSD	2.03	.16	.05
_ln_RMSSD	5.54	.02	.13
pNN50	3.09	.09	.08
HF	2.17	.15	.05
_ln_HF	5.02	.03	.12
LF	2.39	.13	.06
_ln_LF	10.81	.00	.22
SD2	4.78	.04	.11

*Note*. ANCOVA: the first 4 minutes of the first experimental block as the dependent variable, Group as a fixed factor and the 4-minute-long resting period before the experiment as a covariate. Group: group of participants in the Gatekeeper task group and in the Documentary-viewing group.

### Changes in HR and HRV with Time-on-Task

The Time-on-Task analyses focused on the changes in HR and HRV from block 1 to block 5 (see Table 2). The significant Block x Group interactions showed that each cardiac parameter changed differentially in the Gatekeeper task and the Documentary-viewing groups. Simple effect analysis revealed that, compared to the Documentary-viewing group, there was a significant linear decrease in HR in the Gatekeeper task group. In addition, in the Gatekeeper task group, four vagal mediated HRV components, (i.e. RMSSD, _ln_RMSSD, pNN50, and HF) increased linearly from block 1 to block 5, whereas no significant changes were observed in the Documentary-viewing group. _ln_HF, LF, _ln_LF, and SD2 showed significant increases both in the Gatekeeper task group and the Documentary-viewing group, but those changes were always larger in the Gatekeeper group (see also Fig 4).

**Table 2:** Results of *m*ANCOVAs and follow-up simple effects analyses for the changes in heart rate and HRV in Time-on-Task.

Variables	Analysis							
	*mANCOVAs*				*Simple effects analyses*			
	Block effect		Block x Group		Block effect (Gatekeeper task group)		Block effect (Documentary-viewing group)	
	F_(4,152)_	η_*p*_^2^	F_(4,152)_	η_*p*_^2^	F_(4,35)_	η_*p*_^2^	F_(4,35)_	η_*p*_^2^
HR	4.75**	.11	16.83***	.31	25.92***	.75	2.04	.19
RMSSD	4.01*	.10	3.57*	.09	8.96***	.51	1.69	.16
_ln_RMSSD	10.75***	.22	6.34**	.14	13.16***	.60	2.52^m^	.22
pNN50	6.61**	.15	3.56*	.09	6.70***	.43	1.52	.15
HF	2.572^*m*^	.06	2.97*	.07	4.20**	.32	.45	.05
_ln_HF	11.09***	.23	5.35**	.12	11.14***	.56	2.72*	.24
LF	12.14***	.24	2.89*	.07	6.97***	.44	4.98**	.36
_ln_LF	5.61**	.13	3.75*	.09	20.92***	.71	6.85***	.44
SD2	9.81***	.21	5.09**	.12	15.04***	.63	6.96***	.44

*Note*. *m*ANCOVA: Block as a within-subject, Group as a between-subject factor and pre-experiment resting HRV as a covariate; Block: five 15-minute-long intervals (selected in each block) in the Time-on-Task period. Group: group of participants in the Gatekeeper task group and in the Documentary-viewing group

*p < .05

**p < .01

***p < .001, m: *p =* .05 –.06.

In addition, we analyzed whether subjective fatigue *after* the Time-on-Task period was associated with the change in HRV *during* Time-on-Task (i.e. the difference between block 5 and block 1). This showed that participants who displayed a stronger increase in HVR, including vagal-mediated HRV components, also reported more subjective fatigue after the Time-on-Task (RMSSD: r = .51, *p* = .03; HF: r = .50, *p* = .03; LF: r = .47, *p* = .04; SD2: r = .47, *p* = .04).

### Changes in HR and HRV during recovery

Recovery analyses involved the changes in HR and HRV from the last 4 minutes of block 5 to the 4-minute-long resting interval in the break (see Table 3). We found that, during the recovery period, three vagal-mediated HRV components (i.e. RMSSD, _ln_RMSSD, pNN50 and _ln_HF) increased in the Gatekeeper task group. The participants in the Documentary-viewing group did not show increases on those measures. In addition, while participants’ HR in the Gatekeeper task group decreased during the recovery period, no change was found in the Documentary-viewing group.

**Table 3:** Results of ANCOVAs for the changes in heart rate and HRV in recovery.

Variables	Analysis		
	Group effect		
	F_(1,38)_	*p*	η_*p*_^2^
HR	16.40	< .001	.30
RMSSD	6.04	.02	.14
_ln_RMSSD	8.19	< .01	.18
pNN50	13.36	.00	.26
HF	.80	.38	.02
_ln_HF	5.70	.02	.13
LF	.36	.55	.01
_ln_LF	.73	.40	.02
SD2	.60	.44	.02

*Note*. ANCOVA: the 4-minute-long resting period in the break as the dependent variable, Group as a fixed factor and the last 4 minutes of block 5 as covariate; Group: Gatekeeper task group and the Documentary-viewing group.

### Reactivity in HR and HRV after the break

Reactivity analyses after the break involved the changes in HR and HRV from the 4-minute resting interval during the break to the first 4 minutes of the post-break block (see Table 4). The analysis revealed that participants’ HR increased in the Gatekeeper task group, but decreased in the Documentary-viewing group. Of the HRV components, only one vagal-mediated HRV component (_ln_RMSSD) showed a significant reactivity related change after the break: in reactivity, lnRMSSD significantly decreased in the Gatekeeper task group relative to the Documentary-viewing group.

**Table 4:** Results of ANCOVAs for the changes in heart rate and HRV in reactivity after the break.

Variables	Analysis		
	Group effect		
	F_(1,38)_	*p*	η_*p*_^2^
HR	22.99	< .001	.38
RMSSD	3.39	.07	.08
_ln_RMSSD	4.32	.04	.10
pNN50	1.37	.25	.04
HF	1.04	.31	.03
_ln_HF	1.82	.19	.05
LF	1.27	.27	.03
_ln_LF	.58	.45	.02
SD2	1.22	.28	.03

*Note*. ANCOVA: the first 4 minutes of the post-break block as the dependent variable, Group as a fixed factor and the 4-minute-long resting period of the break as covariate. Group: the Gatekeeper task group and the Documentary-viewing group.

### Motivation and inattention: Analysis of post-error cardiac activity, post-error reaction times, and reaction time variability

In the Gatekeeper task, phasic heart rate activity was significantly slower after inaccurate responses compared to after correct responses (F(1,19) = 9.65, *p* < .01, η_*p*_^2^ = .34). The Block x Correctness of Responses interaction, however, was not significant, suggesting that this error-related cardiac activity remained unchanged over Time-on-Task (F(4,76) = 1.13, *p* = .35). Similarly, responses were generally slower in a trial if the response in the previous trial was erroneous (F(1,19) = 44.42, *p* < .001, η_*p*_^2^ = .70), but no significant interaction with Time-on-Task was obtained (F(4,76) = .3, *p* = .78). All in all, these findings seem to indicate that the participants did not strongly decrease in their willingness to do well on the task because their reaction to errors remains relatively stable over time.

Finally, RT variability (i.e. SD / mean)–as a frequently used index of the lapses in attention, or inattention level–was calculated for each block of trial. We found that RT variability significantly increased during the prolonged performance period (F(4,76) = 4.51, *p* < .05., η_*p*_^2^ = .19) suggesting that participants became substantially inattentive during the prolonged performance of the task.

## Discussion

The present study examined the temporal profile of HRV, including the changes related to reactivity, Time-on-Task, and recovery. Our hypotheses were based on previous observations that the activity of the parasympathetic system predicts a wide range of fatigue-vulnerable cognitive functions, including task disengagement [34]. Accordingly, we expected Time-on-Task related changes particularly on those HRV components that are presumed indices of vagal activity [15]. We also analyzed post-response cardiac activity (i.e. heart rate change) and post-error reaction times as presumed correlates of performance monitoring [37, 22].

### Reactivity

Reactivity refers to the change from resting state to working on the task. In the reactivity periods, vagal-mediated HRV measures (i.e. _ln_RMSSD, _ln_HF in the first reactivity and _ln_RMSSD in the second reactivity) decreased more (i.e. significant vagal withdrawal) in the participants who engaged in the Gatekeeper task group than in participants who started to view the documentary. This is in line with our expectation that the parasympathetic system flexibly adapts to the enhanced effort that needs to be mobilized to a cognitively demanding task. Regarding the effect of the LF component, it is relevant to note that this component is affected by sympathetic as well as parasympathetic outflows [57]. Oscillation in LF power is suggested to mainly provide information about blood pressure control, such as baroreflex sensitivity [58–60] found that baroreceptor function was inhibited during cognitive task performance, possibly to allow increases in cardiovascular activity when exerting mental effort. A similar inhibitory process might also the reason for the decreased _ln_LF during reactivity.

The significant increase in HR in the reactivity period of the Gatekeeper task was in line with the notion that voluntary modulated mental effort involves widespread brain activation and is associated with increasing heart rate [61].

### Time-on-Task

Compared to the Document-viewing group, each HRV measure, including the five measures as presumed indices of vagal activity (i.e. RMSSD, _ln_RMSSD, pNN50, HF, _ln_HF), showed a greater increase over time in the participants who engaged in the Gatekeeper task. In line with our hypothesis and previous studies [26, 38, 39], this finding suggests an enhanced vagal inhibition on heart activity with increasing time spent on a fatiguing cognitive task. Note that vagal control on cardiac activity, including HRV level, has been found to be influenced by activity of the locus coeruleus-norepinephrine system (LC-NE system; [62]). The locus coeruleus is a central core in the brain stem that is responsible for the release of norepinephrine in the brain. Higher LC-NE activity is associated with lower parasympathetic influence on HRV. Interestingly, the LC-NE system has been suggested to be less active under fatigue [3, 23, 34]. Such lowered LC-NE system activity is assumed to partly underlie the attentional difficulties that tend to occur under fatigue. Therefore, our findings on the increased vagal-mediated HRV with increasing Time-on-Task fits with the previous research suggesting fatigue-related declines in activation of LC-NE system [23, 34].

Additional correlational analyses also revealed that those participants who reported the most subjective fatigue after the 5 blocks of the Gatekeeper task (i.e. Time-on-Task), were the ones who showed significantly more HF and RMSSD increases. Subjective fatigue, increased parasympathetic activity, as well as lower LC-NE system activation are known to be accompanied with task disengagement [35, 63]. Therefore, the overall pattern of findings on subjective fatigue and HRV seem to converge in the notion that, over time, participants tended to disengage from the Gatekeeper task. Task disengagement, however, is a complex phenomenon, including at least three domains: i) task motivation, ii) concentration, and iii) energetic arousal [64].

First, regarding task motivation, there are reasons to believe that the deliberate motivational aspects of engagement were not strongly diminished. Actual performance remained relatively stable over the five blocks, suggesting that the participants still tried to maintain their performance level despite rising feelings of fatigue and workload. In addition, participants’ heart rate decelerated, and their response slowed down after making erroneous responses. These findings suggest that participants were aware of their inaccurate responses and were still motivated to exert compensatory effort during the Time-on-Task period.

Second, in contrast to motivation, we found evidence that the concentration aspect of task disengagement changed by the Time-on-Task, because participants’ reaction time variability as an index of concentration level showed significant change during the Time-on-Task [65]. This finding makes sense, as previous literature suggest that increased variability in attention is a typical manifestation of fatigue-related effects on performance [66, 67].

Third, Time-on Task related increase in vagal-mediated HRV may be indicative of a lowered energetic arousal, the third aspect of task disengagement. Energetic arousal was proposed by [68] and reflects a continuum in subjective state ranging from tiredness to energy. Low levels of energetic arousal can be manifested in decreased preparation for allocating attentional resources to the task [64] and a decline in vigilance [69]. In addition, based on their investigation of vagal-mediated HRV components [70], reported that higher levels of energetic arousal were accompanied by a shift from activation of the autonomic nervous system to sympathetic dominance. Subsequently, the present findings suggest that participants’ energetic arousal may have also declined during Time-on-Task.

As a function of the time spent on the Gatekeeper task, the low frequency indices and the non-linear SD2 component of HRV increased too. These findings indicate a Time-on-Task related enhancement of baroreceptor sensitivity, which suggests a decline in cognitive effort [71]. This interpretation should be considered with some caution, however, as there are also studies suggesting that LF may not be a robust index of baroreceptor sensitivity [72].

Noteworthy is that many of the HRV components clearly increased in the Gatekeeper group as well as in the Documentary viewing group (i.e. _ln_HF, LF, _ln_LF, and SD2). Thus, HRV is responsive even when individuals are engaged in a cognitively low demanding, non-fatiguing activity for a prolonged period. This highlights the relevance of comparing high versus low demanding conditions in fatigue research in order to examine to what extent HRV and Time-on-Task change as a function of the level of cognitive demand.

### Recovery and reactivity after the break

During the break (after Time-on-Task), participants in the Gatekeeper task group displayed recovery in five HRV measures including vagal-mediated components (RMSSD, _ln_RMSSD, _ln_HF, pNN50). These findings suggests that taking a rest after a cognitively demanding task is accompanied with parasympathetic-mediated relaxation processes. After the break, we could observe vagal withdrawal again when participants started to work on the last block of the Gatekeeper task. In addition, in the Gatekeeper task group, heart rate also showed significant reactivity (i.e. increase) again after the break.

Regarding performance, participants showed a rather strong improvement after the break. One possibility is that such a cognitive improvement might be related to activity in both the task-related and Default Mode Network (DMN) neural connectivity during the break [73]. A higher vagal tone has also been associated with DMN activation in a previous study [74] suggesting that the recovery-related change we found in vagal mediated HRV may belong to those functional changes that contribute to an effective restoration.

### Limitations of the study

The present study can contribute to insight into the nature of fatigue and Time-on-Task related effects because it provides detailed information on the changes in HR and HRV when engaging in a cognitively demanding task for a prolonged period. Nevertheless the study also had at least three limitations to consider when interpreting the results.

First, one of the most important procedural elements of this study was the use of a documentary viewing as a control condition. Identical control conditions have been frequently used in previous fatigue research, but it is important to note that we compared two complex and qualitatively different conditions. Specifically, although the cognitive task was mentally more fatiguing, this was just one of the many factors, which made this task different to documentary viewing. Therefore, the differences we observed between the two conditions may have been derived not only from the difference in fatigue but also from other factors as for example, the perceptual, movement, and affective factors characterized the Gatekeeper task and the documentary viewing differently. Future studies may consider control conditions, which more systematically changed relative to the cognitive task.

Second, in reactivity and recovery, a longer monitoring interval for the calculation of the time domain HRV indices would have been better. On the other hand, we aimed to specifically observe the period when individuals experience a change in cognitive demand, and therefore a longer interval would have extended beyond this psychologically special period. As mentioned above, HRV studies frequently use short monitoring intervals to observe changes related to reactivity and recovery (see e.g. [51, 52]). Third, the explanations about the underlying physiological source of HRV findings would have been benefitted from the monitoring of additional physiological measurements with clear physiological sources (e.g. pupillography, and skin conductance).

### Concluding statements and practical implications

To summarize the main findings of the present study, we showed that Time-on-Task on a dual 2-back task with a game-like character (i.e. the Gatekeeper task) was associated with an elevated level of subjective fatigue and, concurrently, with decreased heart rate as well as increased HRV. Compared to a cognitively less demanding documentary viewing condition, the vagal-mediated HRV components showed a clear differential trend suggesting that the parasympathetic branch of the autonomous nervous system functioning as a relaxation system tends to be activated under increasing mental fatigue. Based on post-error cardiac slowing and post-error RT analyses, we found no evidence for strong motivation deficit in association with increasing Time-on-Task. In addition, similar to HRV, HR was also tended to be changed in all three phases of the study: HR increased in reactivity, and decreased in Time-on-Task, and recovery.

Finally, the findings on HRV in relation to Time-on-Task may have practical implications. Accumulation of fatigue can seriously impact work efficiency and safety. In addition, frequent episodes of acute mental fatigue may increase the risk of developing more chronic forms of fatigue. For these reasons, many technical systems and analytic approaches have been developed to monitor the physiological manifestations of mental fatigue including plenty of studies that suggest that HRV may be particularly suited as a marker of fatigue [75–78]. The current study contributes to this field by suggesting that among the many HRV components, it may especially be the vagal-mediated components of the HRV spectrum that are reliable physiological indicators of operators’ fatigue in different work context.

## Supporting information

S1 File (DOCX)
