# Correction: Enhanced cardiac vagal tone in mental fatigue: Analysis of heart rate variability in Time-on-Task, recovery, and reactivity

**DOI:** 10.1371/journal.pone.0296233

**Published:** 2023-12-20

**Authors:** András Matuz, Dimitri van der Linden, Zsolt Kisander, István Hernádi, Karádi Kázmér, Árpád Csatho

The fifth author’s name is spelled incorrectly. The correct name is: Kázmér Karádi. The correct citation is: Matuz A, van der Linden D, Kisander Z, Hernádi I, Karádi K, Csathó Á (2021) Enhanced cardiac vagal tone in mental fatigue: Analysis of heart rate variability in Time-on-Task, recovery, and reactivity. PLoS ONE 16(3): e0238670. https://doi.org/10.1371/journal.pone.0238670.
