# Supplementary material for: Enhanced cardiac vagal tone in mental fatigue: Analysis of heart rate variability in Time-on-Task, recovery, and reactivity
Source: PLoS One. 2021 Mar 3;16(3):e0238670. doi: 10.1371/journal.pone.0238670 (PMC7928498; doi:10.1371/journal.pone.0238670)
Supplement: S1 File — (DOCX) [file pone.0238670.s001.docx]

**S1 File**

**Enhanced Cardiac Vagal Tone in Mental Fatigue: Analysis of Heart Rate Variability in Time-on-Task, Recovery, and Reactivity**

András Matuz, Dimitri van der Linden, Zsolt Kisander, István Hernádi, Karádi Kázmér, Árpád Csathó*

^*^ Correspondence:

Árpád Csathó

Institute of Behavioral Sciences, Medical School

University of Pécs, Szigeti str. 12, Pécs, 7624, Hungary

e-mail: arpad.csatho@aok.pte.hu

Phone: +(36) 72 536 256

| **Variables** | **Time of Administration of VAS_fatigue_ and** **NASA_TLX_** | | | |
| --- | --- | --- | --- | --- |
|  | *Before ToT* | *After ToT* | *After the break* | *After the post-break task* |
| *Gatekeeper task group* | | | | |
| Subjective Fatigue | 27.40(18.46) | 52.80(15.44) | 49.95(16.94) | 48.40(17.55) |
| Workload |  |  |  |  |
| Mental demand | 11.70(4.76) | 17.10(2.02) |  | 11.40(4.50) |
| Physical demand | 1.75(1.12) | 10.35(4.61) |  | 6.35(4.76) |
| Temporal demand | 10.90(5.56) | 12.05(3.80) |  | 9.00(4.83) |
| Performance | 5.95(2.87) | 11.20(2.57) |  | 9.30(3.26) |
| Effort | 12.40(4.37) | 16.15(3.18) |  | 12.30(4.16) |
| Frustration | 7.75(4.86) | 11.15(4.73) |  | 8.15(5.15) |
|  |  |  |  |  |
| *Documentary-viewing group* | | | | |
| Subjective Fatigue | 37.04(22.11) | 43.71(22.02) | 39.78(21.56) | 41.64(20.83) |
| Workload |  |  |  |  |
| Mental demand | 3.62(2.44) | 6.05(3.41) |  | 4.09(3.53) |
| Physical demand | 1.38(.59) | 3.62(2.63) |  | 3.09(2.53) |
| Frustration | 1.48(1.12) | 2.71(2.30) |  | 3.81(3.91) |

**Table S1.** Descriptive statistics (Mean, (SD)) of the subjective fatigue (VAS_fatigue_) and workload measures (NASA_TLX_) in the Gatekeeper task group and the Documentary-viewing group.

*Note*. ToT: Time-on-Task

**Table S2.** Results of ANOVAs of change scores (i.e. Group effects) for subjective measures in each phase of the experiment.

| **Subjective measures** | **Phases of the experiment** | | | | | |
| --- | --- | --- | --- | --- | --- | --- |
|  | *Time-on-Task period* | | *Break* | | *Post-break block* | |
|  | F_(1,39)_ | η*_p_*^2^ | F_(1,39)_ | η*_p_*^2^ | F_(1,39)_ | η*_p_*^2^ |
| Subjective Fatigue | 14.41*** | .27 | .09 | .00 | .80 | .02 |
| Mental demand | 6.30* | .14 | - | - | 19.81*** | .34 |
| Physical demand | 35.30*** | .48 | - | - | 9.91** | .20 |
| Frustration | 4.22* | .10 | - | - | 10.81** | .22 |

*Note.* ANOVAs of change: ANOVAs were conducted with change scores as dependent variables and Group as a fixed factor. For the Time-on-Task period, change scores were calculated as the difference between the pre-Time-on-Task measurement and the post-Time-on-Task measurement. For the change score in subjective fatigue during the break, the post break measurement was subtracted from the post-Time-on-Task measurement. For the Post-break block, the change scores were calculated as the difference between the post-break measurement and the measurement after the post-break block.

Group: group of participants in the Gatekeeper task group and in the Documentary-viewing group

**p* < .05, ***p* < .01, ****p* < .001

| **Variables** | **Blocks of trials** | | | | | | |  |
| --- | --- | --- | --- | --- | --- | --- | --- | --- |
|  | *Time-on-Task blocks* | | | | | *post-break block* | |  |
|  | B1 | B2 | B3 | B4 | B5 | B6 | |  |
| *Accuracy* |  | | | | | |  | |
| visual target | 0.85(0.10) | 0.87 (0.08) | 0.86(0.13) | 0.85(0.09) | 0.83(0.12) | 0.86(0.10) | |  |
| auditory target | 0.82(0.10) | 0.85(0.09) | 0.87(0.09) | 0.87(0.11) | 0.85(0.13) | 0.85(0.15) | |  |
| dual target | 0.93(0.07) | 0.93(0.08) | 0.94(0.07) | 0.92(0.09) | 0.90(0.10) | 0.95(0.08) | |  |
| no target | 0.75(0.09) | 0.75(0.13) | 0.77(0.13) | 0.78(0.14) | 0.77(0.15) | 0.83(0.13) | |  |
|  | | | | | | |  | |
| *RT* |  | | | | | |  | |
| visual target | 1.07(0.22) | 0.97(0.24) | 0.94(0.28) | 0.93(0.26) | 0.93(0.27) | 0.90(0.24) | |  |
| auditory target | 1.10(0.24) | 1.03(0.23) | 0.97(0.24) | 0.93(0.23) | 0.94(0.21) | 0.91(0.20) | |  |
| dual target | 0.98(0.24) | 0.87(0.26) | 0.84(0.26) | 0.80(0.23) | 0.81(0.24) | 0.78(0.23) | |  |
| no target | 1.22(0.25) | 1.15(0.26) | 1.08(0.27) | 1.05(0.25) | 1.02(0.24) | 0.98(0.20) | |  |
|  | | | | | | |  | |
| *d*’ |  | | | | | |  | |
| visual target | 1.83(0.51) | 1.99(0.75) | 2.11(0.95) | 2.08(0.87) | 1.99(1.02) | 2.27(0.91) | |  |
| auditory target | 1.70(0.61) | 1.87(0.72) | 2.07(0.84) | 2.18(1.07) | 2.11(1.11) | 2.31(1.11) | |  |
| dual target | 2.29(0.63) | 2.43(0.87) | 2.63(0.97) | 2.49(1.12) | 2.40(1.14) | 2.89(0.89) | |  |
|  | | | | | | |  | |
| *Z_d’_- Z_RT_* |  | | | | | |  | |
| visual target | -0.79(1.00) | -0.26(1.12) | 0.01(1.47) | 0.00(1.50) | -0.09(1.58) | 0.34(1.20) | |  |
| auditory target | -1.05(1.17) | -0.59(1.02) | -0.15(1.25) | 0.10(1.45) | 0.01(1.41) | 0.33(1.44) | |  |
| dual target | 0.04(1.17) | 0.63(1.22) | 0.94(1.51) | 0.94(1.52) | 0.83(1.55) | 1.44(1.30) | |  |

**Table S3.** Descriptive statistics (Mean, (SD)) of four performance measures of the Gatekeeper task in the Time-on-Task blocks (B1 – B5) and the post-break block (B6).

Note. *Z_d’_- Z_RT_*: Combined score; *d*’: target sensitivity

**Table S4.** Descriptive statistics (Mean. SD) for Heart rate and HRV variables in the Gatekeeper task group.

| **Variables** |  | **Phases of the experiment** | | | | | | | | | | | |
| --- | --- | --- | --- | --- | --- | --- | --- | --- | --- | --- | --- | --- | --- |
|  |  | *Reactivity* | | | *Time-on-Task* | | | | | *Recovery* | | *Reactivity* | |
|  |  | Resting | B1-first | B1 | | B2 | B3 | B4 | B5 | B5-last | Resting | B6-first | B6 |
|  |  | 4-min | 4-min | 15-min | | 15-min | 15-min | 15-min | 15- min | 4-min | 4-min | 4-min | 15-min |
| HR | M | 79.62 | 87.88 | 86.82 | | 83.21 | 80.97 | 78.77 | 76.91 | 76.27 | 71.00 | 73.73 | 74.30 |
|  | SD | 10.62 | 15.55 | 15.68 | | 13.75 | 12.60 | 11.08 | 10.65 | 10.74 | 10.27 | 10.80 | 10.97 |
| RMSSD | M | 44.49 | 40.29 | 43.12 | | 45.59 | 47.60 | 52.97 | 61.60 | 64.19 | 71.03 | 69.08 | 44.49 |
|  | SD | 20.81 | 28.78 | 30.95 | | 29.38 | 28.61 | 25.13 | 36.68 | 41.99 | 37.79 | 37.69 | 20.81 |
| _ln_RMSSD | M | 3.69 | 3.43 | 3.52 | | 3.63 | 3.71 | 3.86 | 3.97 | 4.00 | 4.13 | 4.10 | 4.07 |
|  | SD | 0.50 | 0.80 | 0.76 | | 0.64 | 0.57 | 0.49 | 0.55 | 0.58 | 0.55 | 0.54 | 0.61 |
| pNN50 | M | 22.02 | 17.91 | 19.09 | | 21.37 | 22.35 | 25.50 | 29.31 | 31.26 | 36.26 | 37.29 | 34.95 |
|  | SD | 15.77 | 18.15 | 18.52 | | 18.18 | 17.96 | 16.65 | 17.25 | 19.07 | 21.24 | 21.49 | 21.40 |
| HF | M | 953.35 | 1041.20 | 921.59 | | 1020.87 | 1180.69 | 1224.65 | 1522.28 | 1619.65 | 2029.75 | 1866.95 | 2034.54 |
|  | SD | 841.29 | 1609.18 | 1125.08 | | 1174.13 | 1503.75 | 1108.74 | 1500.06 | 1468.72 | 1662.40 | 1700.73 | 2115.99 |
| _ln_HF | M | 6.40 | 5.99 | 6.11 | | 6.33 | 6.48 | 6.72 | 6.89 | 6.95 | 7.18 | 7.07 | 7.08 |
|  | SD | 1.09 | 1.59 | 1.37 | | 1.19 | 1.13 | 0.96 | 1.00 | 1.04 | 1.07 | 1.05 | 1.12 |
| LF | M | 1591.18 | 618.25 | 1033.98 | | 1189.52 | 1452.20 | 1893.65 | 2069.67 | 2384.62 | 1962.26 | 1846.26 | 1756.85 |
|  | SD | 1086.62 | 493.89 | 1003.50 | | 844.18 | 959.25 | 1222.09 | 1301.75 | 1799.60 | 1142.50 | 1360.20 | 1032.72 |
| _ln_LF | M | 7.11 | 6.00 | 6.53 | | 6.83 | 7.05 | 7.33 | 7.43 | 7.41 | 7.35 | 7.28 | 7.28 |
|  | SD | 0.82 | 1.08 | 1.01 | | 0.76 | 0.75 | 0.71 | 0.69 | 0.98 | 0.81 | 0.72 | 0.67 |
| SD2 | M | 63.08 | 43.42 | 51.49 | | 57.48 | 63.07 | 70.57 | 75.15 | 76.22 | 80.72 | 71.25 | 74.04 |
|  | SD | 21.32 | 20.74 | 23.96 | | 22.73 | 24.06 | 24.21 | 24.76 | 27.29 | 24.94 | 24.04 | 27.12 |

*Note*. HR: Heart rate; RMSSD: root mean square of successive differences (ms); pNN50: the percent of the number of pairs of adjacent RR intervals differing by more than 50 ms (%); HF: High frequency power (0.15Hz - 0.4 Hz; ms^2^); LF: Low frequency power (0.04Hz – 0.15Hz; ms2); SD2: a measure of the length of the Poincaré could

B1 – B5: time-on-task blocks; B1-first: first 4 minutes of block 1; B5-last: last 4 minutes of block 5; B6-first: first 4 minutes of block 6.

| **Variables** |  | **Phases of the experiment** | | | | | | | | | | | |
| --- | --- | --- | --- | --- | --- | --- | --- | --- | --- | --- | --- | --- | --- |
|  |  | *Reactivity* | | | *Time-on-Task* | | | | | *Recovery* | | *Reactivity* | |
|  |  | Resting | B1-first | B1 | | B2 | B3 | B4 | B5 | B5-last | Resting | B6-first | B6 |
|  |  | 4-min | 4-min | 15-min | | 15-min | 15-min | 15-min | 15- min | 4-min | 4-min | 4-min | 15-min |
| HR | M | 85.10 | 81.76 | 81.43 | | 80.34 | 79.63 | 78.67 | 78.02 | 77.32 | 78.15 | 75.38 | 74.61 |
|  | SD | 12.98 | 12.43 | 11.14 | | 10.31 | 9.91 | 10.32 | 10.70 | 11.05 | 12.95 | 13.08 | 11.50 |
| RMSSD | M | 41.84 | 46.55 | 38.77 | | 42.21 | 42.47 | 44.10 | 44.89 | 45.45 | 41.14 | 50.77 | 51.67 |
|  | SD | 34.27 | 42.72 | 30.62 | | 29.74 | 31.36 | 30.23 | 30.54 | 26.07 | 32.73 | 39.19 | 38.12 |
| _ln_RMSSD | M | 3.42 | 3.49 | 3.38 | | 3.50 | 3.50 | 3.57 | 3.57 | 3.67 | 3.48 | 3.62 | 3.67 |
|  | SD | 0.81 | 0.84 | 0.79 | | 0.77 | 0.75 | 0.69 | 0.74 | 0.54 | 0.70 | 0.86 | 0.81 |
| pNN50 | M | 16.24 | 18.16 | 16.31 | | 17.74 | 17.28 | 19.14 | 19.46 | 21.60 | 16.63 | 22.25 | 22.60 |
|  | SD | 20.71 | 24.64 | 21.23 | | 20.18 | 20.05 | 20.31 | 19.97 | 21.09 | 19.83 | 22.98 | 21.66 |
| HF | M | 925.37 | 1389.67 | 983.61 | | 976.45 | 1036.96 | 1183.03 | 990.14 | 1045.24 | 1100.91 | 1388.29 | 1343.70 |
|  | SD | 1393.61 | 2564.84 | 1549.04 | | 1418.99 | 1625.17 | 1896.21 | 1184.29 | 1469.96 | 2123.16 | 2255.05 | 1903.93 |
| _ln_HF | M | 5.71 | 6.11 | 5.80 | | 6.01 | 6.00 | 6.18 | 6.15 | 6.29 | 5.94 | 6.08 | 6.25 |
|  | SD | 1.60 | 1.66 | 1.61 | | 1.49 | 1.53 | 1.42 | 1.44 | 1.22 | 1.46 | 1.78 | 1.64 |
| LF | M | 2143.84 | 946.68 | 967.30 | | 1382.55 | 1519.08 | 1682.19 | 1561.95 | 1386.64 | 1688.58 | 1340.19 | 1812.47 |
|  | SD | 4357.56 | 757.31 | 733.94 | | 963.54 | 1088.00 | 1272.76 | 838.20 | 895.13 | 1661.06 | 1128.56 | 1495.72 |
| _ln_LF | M | 6.72 | 6.61 | 6.52 | | 6.88 | 6.95 | 7.07 | 7.09 | 7.08 | 6.92 | 6.69 | 7.10 |
|  | SD | 1.36 | .84 | 1.13 | | 1.19 | 1.19 | 1.12 | 1.05 | .62 | 1.08 | 1.48 | 1.26 |
| SD2 | M | 61.24 | 52.95 | 51.96 | | 59.53 | 61.76 | 65.09 | 63.65 | 62.00 | 63.67 | 64.33 | 67.34 |
|  | SD | 40.96 | 27.84 | 24.80 | | 26.07 | 27.45 | 27.99 | 22.33 | 22.69 | 30.18 | 29.10 | 28.90 |

**Table S5.** Descriptive statistics (Mean. SD) for Heart rate and HRV variables in the Documentary-viewing group.

*Note*. HR: Heart rate; RMSSD: root mean square of successive differences (ms); pNN50: the percent of the number of pairs of adjacent RR intervals differing by more than 50 ms (%); HF: High frequency power (0.15Hz - 0.4 Hz; ms^2^); LF: Low frequency power (0.04Hz – 0.15Hz; ms2); SD2: a measure of the length of the Poincaré could

B1 – B5: Time-on-Task blocks; B1-first: first 4 minutes of block 1; B5-last: last 4 minutes of block 5; B6-first: first 4 minutes of block 6.

**Table S6**. Results of ANOVAs of change scores (i.e. Group effects) for the changes in Heart Rate and HRV in Reactivity and Recovery.

| **Variables** | **Group effects in three phases of the experiment** | | | | | | | | |
| --- | --- | --- | --- | --- | --- | --- | --- | --- | --- |
|  | *Reactivity* | | | *Recovery* | | | *Reactivity after the break* | | |
|  | F_(1,39)_ | *p* | η*_p_*^2^ | F_(1,39)_ | *p* | η*_p_*^2^ | F_(1,39)_ | *p* | η*_p_*^2^ |
| HR | 7.09 | <.001 | .50 | 16.74 | <.001 | .30 | 26.91 | <.001 | .41 |
| RMSSD | 1.98 | .17 | .05 | 4.75 | .04 | .11 | 4.68 | .04 | .11 |
| _ln_RMSSD | 5.65 | .02 | .13 | 7.69 | .01 | .17 | 3.75 | .06 | .09 |
| pNN50 | 3.14 | .08 | .08 | 12.00 | <.01 | .24 | 2.22 | .14 | .05 |
| HF | 1.04 | .31 | .03 | .99 | .33 | .03 | 1.81 | .19 | .04 |
| _ln_HF | 6.25 | .02 | .14 | 4.42 | .04 | .10 | 1.60 | .21 | .04 |
| LF | .60 | .81 | .00 | 2.74 | .11 | .07 | .29 | .59 | .01 |
| _ln_LF | 11.46 | <.01 | .23 | .12 | .73 | .00 | .36 | .55 | .01 |
| SD2 | 3.16 | .08 | .08 | .34 | .56 | .01 | 3.33 | .08 | .08 |

*Note.* ANOVAs of change: ANOVAs were conducted with change scores as dependent variables and Group as a fixed factor. For change scores in reactivity, the pre-experiment HRV was subtracted from the HRV in the first 4 minutes of the Time-on-Task period. For change scores in recovery, the HRV in the last 4 minutes of the Time-on-Task period was subtracted from the HRV during the break. For change scores in reactivity after the break, the HRV during the break was subtracted from the HRV in the first 4 minutes of the post-break block.

Group: group of participants in the Gatekeeper task group and in the Documentary-viewing group.

**Results for HR and HRV in the Time-on-Task with variable block intervals**

The duration of the blocks in the Time-on-Task period depended on participants’ reaction time (each lasted about 18 minutes). Therefore, for the analyses reported in the main text, we selected a 15-minute-long interval in middle of each block. Here, however, we report the results for analyses performed on the total block duration. In this way, the blocks contained the same number of trials in the Gatekeeper task group, but were moderately variable in duration. These results also support the hypothesis that vagal-mediated HRV increases with Time-on-Task in the Gatekeeper task group, but shows no, or smaller magnitude of change in the Documentary-viewing group.

| **Variables** | **Analysis** | | | | | | | |
| --- | --- | --- | --- | --- | --- | --- | --- | --- |
|  | *mANCOVAs* | | | | *Simple effects analyses* | | | |
|  | Block effect | | Block x Group | | Block effect  (Gatekeeper task group ) | | Block effect (Documentary-viewing group) | |
|  | F_(4,152)_ | η*_p_*^2^ | F_(4,152)_ | η*_p_*^2^ | F_(4,35)_ | η*_p_*^2^ | F_(4,35)_ | η*_p_*^2^ |
| HR | 4.74** | .11 | 18.26*** | .33 | 28.14*** | .76 | 2.35^m^ | .21 |
| RMSSD | 3.35* | .08 | 4.86** | .11 | 9.13*** | .51 | 1.71 | .16 |
| _ln_RMSSD | 11.32*** | .23 | 7.09*** | .16 | 13.86*** | .61 | 3.54* | .29 |
| pNN50 | 7.20*** | .16 | 4.25* | .10 | 7.23*** | .45 | 1.17 | .12 |
| HF | 1.13 | .03 | 2.23^m^ | .05 | 5.39** | .38 | .61 | .07 |
| _ln_HF | 5.96** | .14 | 4.70** | .11 | 10.63*** | .55 | 2.35^m^ | .21 |
| LF | 23.02*** | .38 | 10.46*** | .22 | 24.83*** | .74 | 3.34* | .28 |
| _ln_LF | 2.89* | .07 | 3.45* | .08 | 19.45*** | .69 | 6.96*** | .44 |
| SD2 | 7.28*** | 16 | 5.47** | .13 | 15.33*** | .64 | 6.09** | .41 |

**Table S7.** The results of *m*ANCOVAs and follow-up simple effects analyses for Heart rate and HRV for the time-on-task phase including blocks with variable duration.

*Note*. *m*ANCOVA: Block as a within-subject, Group as a between-subject factor and pre-experiment resting HRV as a covariate

Block: blocks of trials (B1-B5) in the Time-on-Task period.

Group: group of participants in the Gatekeeper task group and in the Documentary-viewing group

**p* < .05, ***p* < .01, ****p* < .001, m*: p <* 0.09

**Results for the HRV normalized with average RR interval**

Recently, there have been a growing number of studies suggesting that heart rate variability should be normalized with respect to average RR interval (e.g. Sacha, 2014; Sacha & Pluta, 2008; Sacha, 2013; Quintana & Heathers, 2014). The reason of the suggestion for this mathematical correction is the non-linear association between heart rate and R-R intervals. More specifically, the same magnitude of change in heart rate has a much higher effect on RR variability when the average heart rate is slow than when the heart rate is fast (see e.g. figure 1 in Sacha, 2013). Therefore, it has been suggested that the calculated HRV should be divided by the average RR interval (i.e. HRV/avRR, or HRV/avRR^2^ for frequency domain components; see e.g. Sacha, 2014). Although this mathematical correction is still not widely used, we performed analyses also on RR normalized HRV data to test whether the results of our main analysis are robust to such a correction procedure. Importantly, we corrected only those HRV indices which were already analysed in previous studies with this correction procedure: RMSSD, absHF, absLF. Please notice that the conclusions for RMSSD and HF (two vagal mediated HRV indices) remained the same as in the main analysis. For LF we, however, found no significant Block x Group interactions suggesting that the results of LF HRV might depend strongly on average heart rate.

**Table S8.** Results of ANCOVAS and *m*ANCOVAs for the changes in RR normalized HRV in Reactivity, Time-on-Task, and Recovery.

| **Variables** | **Analysis** | | | | | |
| --- | --- | --- | --- | --- | --- | --- |
|  | *Group effect* | | *Block effect* | | *Block x Group* | |
|  | F_(1,38)_ | η*_p_*^2^ | F_(4,152)_ | η*_p_*^2^ | F_(4,152)_ | η*_p_*^2^ |
| *Reactivity* |  |  |  |  |  |  |
| RMSSD | .85 | .02 | - | - | - | - |
| HF | .22 | .01 | - | - | - | - |
| LF | 1.50 | .04 | - | - | - | - |
| *Recovery* |  |  |  |  |  |  |
| RMSSD | 3.89^m^ | .09 | - | - | - | - |
| HF | .07 | .00 | - | - | - | - |
| LF | 1.90 | .05 | - | - | - | - |
| *Reactivity after break* |  |  |  |  |  |  |
| RMSSD | .54 | .01 | - | - | - | - |
| HF | .01 | .00 | - | - | - | - |
| LF | 2.23 | .06 | - | - | - | - |
|  |  |  |  |  |  |  |
| *Time-on-Task* |  |  |  |  |  |  |
| RMSSD | 1.57 | .04 | 4.89** | .11 | 3.38* | .08 |
| HF | .65 | .02 | 4.73** | .11 | 2.65^m^ | .07 |
| LF | .84 | .02 | 8.50*** | .18 | 1.80 | .05 |

*Note*. Block: blocks of trials (B1-B5) in the Time-on-Task period

Group: group of participants in the Gatekeeper task group and in the Documentary-viewing group

Please, note that, in contrast to reactivity and recovery analyses, the analysis of Time-on-Task related changes was performed by mANCOVAs, thus Block main effect and Block x Group interaction effects were obtained as well.

**p* < .05, ***p* < .01, ****p* < .001; m*: p <* .06

**Table S9**. Results of ANOVAs of change scores for the changes in RR normalized HRV in Reactivity and Recovery.

| **Variables** | **Three phases of the experiment** | | | | | | | | |
| --- | --- | --- | --- | --- | --- | --- | --- | --- | --- |
|  | *Reactivity* | | | *Recovery* | | | *Reactivity after the break* | | |
|  | F_(1,39)_ | p | η*_p_*^2^ | F_(1,39)_ | p | η*_p_*^2^ | F_(1,39)_ | p | η*_p_*^2^ |
| RMSSD | .79 | .38 | .02 | 2.78 | .10 | .07 | 1.60 | .21 | .04 |
| HF | .09 | .77 | .00 | .09 | .76 | .00 | .24 | .63 | .01 |
| LF | .17 | .69 | .00 | 5.44 | .03 | .12 | 1.91 | .18 | .05 |

*Note.* ANOVAs of change: ANOVAs were conducted with change scores as dependent variables and Group as a fixed factor. For change scores in reactivity, the pre-experiment HRV was subtracted from the HRV in the first 4 minutes of the Time-on-Task period. For change scores in recovery, the HRV in the last 4 minutes of the Time-on-Task period was subtracted from the HRV during the break. For change scores in reactivity after the break, the HRV during the break was subtracted from the HRV in the first 4 minutes of the post-break block.

Group: group of participants in the Gatekeeper task group and in the Documentary-viewing group.
